# Study protocol of a single-arm phase 2 study evaluating the preventive effect of topical hydrocortisone for capecitabine-induced hand-foot syndrome in colorectal cancer patients receiving adjuvant chemotherapy with capecitabine plus oxaliplatin (T-CRACC study)

**DOI:** 10.1186/s12876-022-02411-w

**Published:** 2022-07-14

**Authors:** Yohei Iimura, Naoki Furukawa, Masaaki Ishibashi, Yuka Ahiko, Taro Tanabe, Susumu Aikou, Dai Shida, Masanori Nojima, Seiichiro Kuroda, Narikazu Boku

**Affiliations:** 1 Department of Pharmacy, The IMSUT Hospital, The Institute of Medical Science, The University of Tokyo, 4-6-1, Shirokanedai, Minato-ku, Tokyo, 108-8639 Japan; 2 Division of Frontier Surgery, The Institute of Medical Science, The University of Tokyo, 4-6-1, Shirokanedai, Minato-ku, Tokyo, 108-8639 Japan; 3 The Department of Surgery, The IMSUT Hospital, The Institute of Medical Science, The University of Tokyo, 4-6-1, Shirokanedai, Minato-ku, Tokyo, 108-8639 Japan; 4 Center for Translational Research, The Institute of Medical Science, The University of Tokyo, 4-6-1, Shirokanedai, Minato-ku, Tokyo, 108-8639 Japan; 5 Department of Oncology and General Medicine, The Institute of Medical Science Hospital, The University of Tokyo, 4-6-1, Shirokanedai, Minato-ku, Tokyo, 108-8639 Japan

**Keywords:** Capecitabine, Hand foot syndrome, Medium class topical corticosteroid, Prevention

## Abstract

**Backgrounds:**

Clinical evidence of the preventive effectiveness of medium-class topical corticosteroids for capecitabine-induced hand foot syndrome (HFS) is limited. Although the pathogenesis and mechanism of HFS are unclear, inflammatory reactions are thought to be involved in HFS development. This study aimed to evaluate the preventive effect of medium-class topical corticosteroids (hydrocortisone butyrate 0.1% topical therapy) for capecitabine-induced HFS in patients with colorectal cancer receiving adjuvant chemotherapy with capecitabine plus oxaliplatin.

**Methods:**

This is a single-center, single-arm, phase 2 study. Patients with colorectal cancer scheduled to receive adjuvant chemotherapy with capecitabine plus oxaliplatin are enrolled, and topical hydrocortisone butyrate 0.1% is applied prophylactically in addition to standard moisturizing therapy. The primary endpoint is the incidence of grade ≥ 2 HFS within three months. The secondary endpoints are the time to onset of HFS, rates of dose reduction, schedule delay, discontinuation caused by capecitabine-induced HFS, and other adverse events. All adverse events are evaluated by clinical pharmacists and attending physicians.

**Discussion:**

This study is expected to contribute to the establishment of new supportive care for preventing HFS, not only for colorectal cancer patients receiving adjuvant chemotherapy, but also for various cancer patients receiving capecitabine-based chemotherapy.

*Trial registration*: This trial was registered in the Japan Registry of Clinical Trials (jRCT) as jRCTs031220002. Registered 5 April 2022, https://jrct.niph.go.jp/search

*Protocol version* V.1.0, 16 February 2022.

## Background

Hand-foot syndrome (HFS) is a major side effect of capecitabine and often impairs the patient's quality of life (QOL) [1]. In randomized controlled trials (RCT) of chemotherapy for colorectal cancer, the incidence of capecitabine-induced HFS was reported to be approximately 30–80% in all grades [2–4], but 90% or more in the real world [5]. Severe HFS presents as swelling, blisters, desquamation, and ulcers, which cause interruptions, schedule delays, dose reductions, and discontinuation of capecitabine.

Two treatment options are recommended for adjuvant chemotherapy of colorectal cancer [6]: oral capecitabine plus intravenous oxaliplatin (CAPEOX regimen) and intravenous 5-fluorouracil and leucovorin plus oxaliplatin (FOLFOX regimen). Although the CAPEOX regimen has a shorter infusion time, its use in clinical practice is limited by the fear of HFS. In addition, HFS affects self-adherence [7]. In particular, the dose intensity of capecitabine affects the outcomes of adjuvant chemotherapy for colon cancer [8]. Thus, HFS management is important not only for the maintenance of patients’ QOL but also for ensuring the treatment efficacy of chemotherapy using capecitabine [9].

Moisturizing and avoiding local pressure [10–12] are generally recognized as standard management methods for preventing capecitabine-induced HFS. Moreover, the effectiveness of exfoliating agents [13, 14], celecoxib [15, 16], and pyridoxine [17] for HFS has been reported. However, there are no established methods for the prevention of capecitabine-induced HFS based on high-level evidence, expect for treatment modification (interruption and/or dose reduction) [18].

The mechanism of HFS involves inhibition of the proliferation of skin basal cells, secretion of drugs from the eccrine sweat glands, involvement of drug degradation products [9, 19], and an inflammatory response caused by IL-1a, IL-1b, IL-6, and reactive oxygen species [20]. Corticosteroids exert anti-inflammatory effects by inhibiting the release of chemical mediators. Oral dexamethasone (8 mg/day, followed by tapering) was administered to patients with pegylated liposomal doxorubicin-induced palmar-plantar erythrodysesthesia in a prospective study [21], which reported that patients receiving dexamethasone could receive treatment without dose modification, but those without dexamethasone administration required schedule delays or dose reductions. Other case reports have suggested that corticosteroids might be effective against cytarabine-and vinorelbine-induced HFS [22, 23]. Similarly, the mechanism of epidermal growth factor receptor (EGFR) inhibitor-related skin rash is assumed to be associated with inflammatory reactions [24–26]. The preventive effect of topical corticosteroids on EGFR inhibitor-related skin rashes has been reported [27, 28]. Based on this evidence, it is expected that topical corticosteroids may prevent the reduction in dose intensity of chemotherapy induced by HFS and improve patients’ quality of life during the adjuvant chemotherapy period. However, no study has clearly suggested the preventive or therapeutic effects of topical corticosteroids for capecitabine-related HFS.

Hence, we conducted a single-center, single-arm, interventional study to evaluate the preventive effect of add-on therapy with medium-class topical corticosteroids (hydrocortisone butyrate 0.1% topical therapy) for capecitabine-induced HFS in the adjuvant chemotherapy of colorectal cancer patients.

We evaluate the preventive effect of 0.1% topical hydrocortisone butyrate therapy for capecitabine-induced HFS in colorectal cancer patients receiving adjuvant chemotherapy with capecitabine and oxaliplatin.

## Methods

### Study design

This trial is a single-center, single-arm, phase 2 study. This protocol was reviewed and approved by the certified Clinical Research Review Board of the University of Tokyo (approval number: 2021512SP). This clinical trial was conducted in compliance with the Clinical Trials Act in Japan and registered in the Japan Registry of Clinical Trials (jRCT) as jRCTs031220002.

### Subjects

The inclusion criteria are: (i) curatively resected and histologically confirmed colorectal adenocarcinoma, (ii) pathological stage II or III, (iii) scheduled to receive adjuvant chemotherapy with capecitabine plus oxaliplatin, (iv) age ≥ 18 years old, (v) ECOG performance status of 0 to 1, (vi) no prior chemotherapy or radiation therapy, (vii) adequate organ function, and (viii) provision of written informed consent. In contrast, the exclusion criteria are: (i) bacterial/fungal/spirochete/virus skin infections, (ii) eczema otitis externa with perforations in the eardrum, (iii) ulcers (excluding Behçet’s disease), second-degree or deeper burns/frostbite, (iv) other skin disease, and (v) a history of hypersensitivity to local hydrocortisone.

### Adjuvant chemotherapy

Adjuvant chemotherapy with capecitabine plus oxaliplatin comprise a 2-h intravenous infusion of oxaliplatin (130 mg/m^2^) on day 1 and oral administration of capecitabine (1000 mg/m^2^, twice daily) from the evening of day 1 to the morning of day 15, repeat every 3 weeks (21 days) for eight or four cycles according to the physician’s discretion and patient’s consent. Dose interruption, delay, reduction, and discontinuation are allowed, if necessary, at the attending physician’s discretion. In principle, capecitabine dose can be reduced by 20% up to 2 levels. In a previous study, the control group was administered celecoxib as the study drug, and the number of cycles to the development of grade 1 and 2 HFS was 4.336 [16]. In addition, the cumulative dose of capecitabine, which causes grade 1 or higher HFS in more than 80% of patients, is 100,000 mg/m^2^ [5]. Therefore, the minimum cumulative dosage of capecitabine in the present study, considering the dose reduction of the drug, should, in principle, be 100,000 mg/m^2^ to complete 4 cycles or more.

### Intervention

**Fig. 1:**
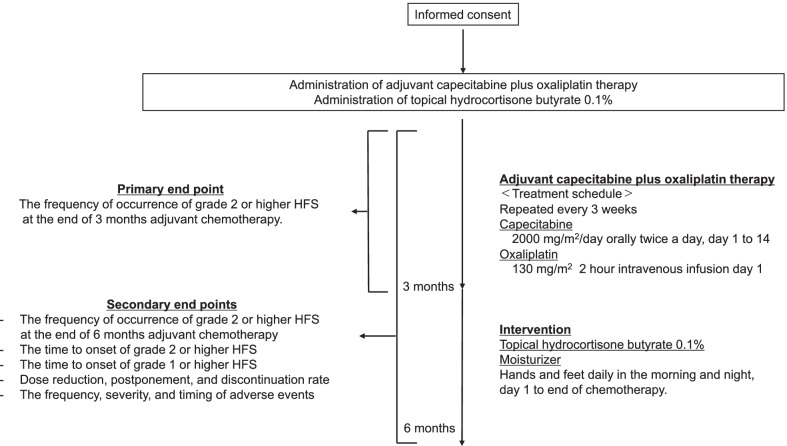
Study scheme. *HFS* hand foot syndrome. Topical hydrocortisone butyrate 0.1% and standard moisturizing therapy are applied to the hands and feet daily in the morning and evening, started on day 1 and continued until the end of adjuvant chemotherapy. To keep patients’ self-adherence, all patients received standard self-care education at the start of the chemotherapy, and is confirmed the amount of topical hydrocortisone butyrate 0.1% used regularly by clinical pharmacists. Clinical pharmacists regularly educate the patients to improve self-adherence to intervention protocols

Topical hydrocortisone butyrate 0.1% and standard moisturizing therapy are applied to the hands and feet daily in the morning and evening. One fingertip unit (1 fingertip unit provides 0.5 g) of topical hydrocortisone butyrate 0.1% is applied to both palms and feet for each application, only once in the morning and once in the evening. This preventive treatment is started on day 1 and continued until the end of adjuvant chemotherapy (Fig. 1). All patients received standard self-care education (ex. keeping clean, moisturizing, wearing gloves, avoiding strenuous exercise, avoiding pressure, and sun protection) at the start of the chemotherapy, and is confirmed the amount of topical hydrocortisone butyrate 0.1% used regularly by clinical pharmacists. Clinical pharmacists regularly educate the patients to improve self-adherence to intervention protocols.

### Criteria for discontinuing interventions

Interventions for the following patients will be discontinued. (i) Withdrawal of consent, (ii) Discontinuation of CAPEOX regimen, (iii) Occurrence of serious adverse events induced by topical hydrocortisone butyrate 0.1%, (iv) Administration of prohibited therapy and drugs. For participants who discontinue or deviate from intervention protocols, optimal medical care without study drugs is guaranteed.

### Prohibited therapy and drugs

The following treatments or concomitant use of drugs are prohibited. (i) Cancer treatment other than CAPEOX regimen, (ii) Administration of other topical corticosteroids for hands or feet, (iii) Administration of celecoxib, (iv) Administration of pyridoxine.

### Evaluation

Patient symptoms and laboratory tests are checked at every visit (Table 1), and the severity of HFS and other adverse events are assessed by the clinical pharmacist and the attending physician, referring to the self-report of adverse events (Tables 2, 3). Adverse events are evaluated based on laboratory parameters and according to the National Cancer Institute Common Terminology Criteria for Adverse Events version 5.0. Evaluators will be provided regular training to promote data quality.

**Table 1:** The assessment schedule

		Chemotherapy (cycles)							
	Pre-treatment	1	2	3	4	5	6	7	8
Performance status	•	•	•	•	•	•	•	•	•
Blood test	•	•	•	•	•	•	•	•	•
Hand foot syndrome		•	•	•	•	•	•	•	•
Adverse events		•	•	•	•	•	•	•	•
Self report (adverse evens)		•	•	•	•	•	•	•	•
Self report (self-adherence)		•	•	•	•	•	•	•	•
Dose of chemotherapy		•	•	•	•	•	•	•	•
Postponement of chemotherapy		•	•	•	•	•	•	•	•
Discontinuation of chemotherapy		•	•	•	•	•	•	•	•

**Table 2:** Self-report of adverse events 1

Side effect self-report 1								
Name								
Date								
	Grade	/	/	/	/	/	/	/
*Stomatitis*								
I have stomatitis but it does not hurt	1							
Stomatitis hurts a little, but I can eat more than half	2							
I can hardly eat because of stomatitis	3							
*Hand foot syndrome*								
Red and swollen, but no pain	1							
There is pain, but it does not interfere with daily life	2							
It is painful and interferes with daily life	3							
*Pigmentation*								
There is pigmentation in limited areas such as fingertips	1							
There is systemic pigmentation	2							
*Peripheral neuropathy*								
There is some numbness, but it does not affect the operation	1							
It is difficult to act due to numbness, but it does not interfere with daily life	2							
Numbness interferes with daily life	3							
I have no sensation	4							
*Peripheral sensory impairment*								
I react instantly to hypersensitivity when I come in contact with cold things	1							
Contact with cold objects sustains a hypersensitive reaction, but is painless	2							
Contact with cold objects causes persistent hypersensitivity and pain	3							
< Please enclose the affected area >

**Table 3:** Self-report of adverse events 2

Side effect self-report 2								
Name								
Date								
	Grade	/	/	/	/	/	/	/
*Nausea*								
Tolerable	1							
If I use anti-nausea drugs, I can manage to eat	2							
I can hardly eat because of nausea	3							
*Anorexia*								
I have a slight loss of appetite	1							
I can eat somehow	2							
I can hardly eat	3							
*Malaise*								
I am a little tired, but it does not interfere with my daily life	1							
I often lie down	2							
I lie down more often than I am awake	3							
Bedridden all day long	4							
*Diarrhea*								
Increased defecation frequency less than 4 times a day compared to usual	1							
Increased defecation frequency 4–6 times a day compared to usual	2							
Increased defecation frequency more than 7 times a day compared to usual	3							
If you have any other symptoms of concern, please write them down								

### Endpoints

The primary endpoint is the incidence of grade ≥ 2 HFS within 3 months of the start of adjuvant chemotherapy. The secondary endpoints are: i) incidence of grade ≥ 2 HFS within 6 months of the start of adjuvant chemotherapy, ii) time to onset of grade ≥ 2 HFS, iii) time to onset of any grade HFS, and iv) incidence of dose reduction, schedule delay, and discontinuation rate of capecitabine caused by any grade of HFS.

### Sample size calculation

For the primary endpoint, the incidence of grade ≥ 2 HFS is calculated among the enrolled patients, excluding those whose adjuvant chemotherapy would be discontinued within 3 months due to recurrence or adverse events other than grade ≥ 2 HFS. In previous RCTs investigating the preventive effect of HFS, the incidence of grade ≥ 2 HFS in the control group without preventive measures was reported to be approximately 40% [15, 16, 29, 30]. It is assumed that hydrocortisone butyrate 0.1% (external application) would suppress grade ≥ 2 HFS within 3 months by 15%, corresponding to the expected incidence of 25% and threshold of 40%. The power was set at 70%, considering feasibility. The power is the probability that the 90% WALD upper confidence limit is below the threshold incidence rate with continuity correction, and the minimum number of patients exceeding 70% is 39 (one-sided α = 0.1). Therefore, considering that some patients were excluded from the primary analysis, the target number of enrolled patients was planned to be 50. The above calculations were performed using SAS Studio 3.8.

### Implementation

NB will generate the allocation sequence, enrol participants, and assign participants to interventions. Clinical pharmacists will support them.

## Discussion

At present, there are no established methods for preventing capecitabine-induced HFS. Moisturizing and avoiding local pressure are widely used to prevent capecitabine-induced HFS in clinical practice because of their low risk of adverse events, although their efficacy has not been proven in clinical trials. Based on the pathogenic mechanism of HFS and previous one prospective study [21] and two case reports [22, 23], we believe that medium-class topical corticosteroids is effective for preventing HFS. This single-center, single-arm, intervention study evaluate the effect of medium-class topical corticosteroids (hydrocortisone butyrate 0.1% topical therapy) added to the present standard care to prevent capecitabine-induced HFS in adjuvant chemotherapy.

The prevention of capecitabine-induced HFS using prophylactic agents, such as exfoliating agents [13, 14], celecoxib [15, 16], and pyridoxine [17], has been reported. Exfoliating agents, which are often used to avoid local pressure for hand-foot reactions induced by other anti-tumor agents, such as sunitinib, sorafenib, and regorafenib, are sometimes irritating and may enhance the symptoms of HFS. In two studies using celecoxib as a preventive drug [15, 16], the frequency of grade ≥ 2 HFS was reduced by 20% or more. However, nonsteroidal anti-inflammatory drugs, including celecoxib, can induce cardiovascular adverse events [31, 32], and long-term administration should be avoided. Therefore, celecoxib is not recommended as a preventive option for HFS in clinical practice. The prophylactic effect of pyridoxine for HFS has been reported [33, 34]. However, the efficacy of pyridoxine is controversial and has been proven to be negative in multiple meta-analyses and RCTs [17, 35–38]. It is important to use agents with a low risk of adverse events to prevent capecitabine-induced HFS.

Topical corticosteroids have some advantages over oral agents owing to their low risk of adverse events. Continued oral steroids can induce infections and should not be administered to patients on chemotherapy. Topical steroids have a low risk of infection because they do not act systemically, and it is needless to consider drug-drug interactions with cytochrome P450 3A4. In addition, topical corticosteroids can be applied at the same time as the moisturizer, which ensures self-adherence.

In the present study, patients are administered medium-class corticosteroids. Stronger topical corticosteroids may increase the risk of local fungal infections and skin atrophy. Therefore, medium-class corticosteroids with low risk of infection can be used safely to prevent HFS.

The preventive effect of hydrocortisone on HFS is expected to improve the QOL for patients on CAPEOX regimen and broaden the choice of adjuvant chemotherapy. This study is expected to contribute to the establishment of new supportive care for preventing HFS, not only for colorectal cancer patients on adjuvant chemotherapy, but also for various cancer patients receiving capecitabine-based chemotherapy.

This study has several limitations. First, it is a single-arm study and not a comparative study. Second, because the study participants receive adjuvant chemotherapy and the evaluation period is as short as 3 months, long-term efficacy and adverse events of topical corticosteroids cannot be evaluated. Based on this study, we will plan an RCT that verifies the preventive effect of topical corticosteroids for HFS, regardless of cancer type, combined agents, or treatment settings.

## Conclusions

We believe that these findings will contribute more evidence on supportive care that is useful not only for adjuvant chemotherapy but also for various cancer treatments that induce HFS. This protocol was established based on the pathogenic mechanism of HFS and previous one prospective study [21] and two case reports [22, 23].

## Data Availability

The data that support the findings of this study are available from the corresponding author, YI, upon reasonable request.

## Abbreviations

CAPEOX: Capecitabine plus intravenous oxaliplatin
EGFR: Epidermal growth factor receptor
FOLFOX: 5-Fluorouracil and leucovorin plus oxaliplatin
HFS: Hand-foot syndrome
jRCT: Japan Registry of Clinical Trials
QOL: Quality of life
RCT: Randomized controlled trial
